# Evaluation of the efficacy of EU-TIRADS and ACR-TIRADS in risk stratification of pediatric patients with thyroid nodules

**DOI:** 10.3389/fendo.2022.1041464

**Published:** 2022-11-22

**Authors:** Gerdi Tuli, Jessica Munarin, Mariapia Scollo, Francesco Quaglino, Luisa De Sanctis

**Affiliations:** ^1^ Department of Health and Pediatric Sciences, University of Turin, Turin, Italy; ^2^ Department of Pediatric Endocrinology, Regina Margherita Children’s Hospital, Turin, Italy; ^3^ Department of Public Health and Pediatrics, University of Turin, Turin, Italy; ^4^ Department of General Surgery, "Maria Vittoria" Hospital Azienda Sanitaria Locale (ASL) Città di Torino, Turin, Italy

**Keywords:** thyroid nodule, pediatric age, ACR-TIRADS, EU-TIRADS, diagnostic performance, thyroid nodules outcome

## Abstract

**Background:**

Pediatric thyroid nodules have a lower prevalence but a higher rate of malignancy (ROM) than those in adults. Ultrasound features suspected of malignancy lead to fine needle aspiration biopsy (FNAB) and subsequent cytological determination, upon which management is decided. Based on the characteristics of ultrasound, to standardize clinician decisions and avoid unnecessary FNAB, the European Thyroid Association and the American Radiology College have established guidelines for Thyroid Imaging, Reporting and Data System (EU-TIRADS and ACR-TIRADS) for ROM stratification of thyroid nodules. The aim of this study is to evaluate the diagnostic performance of ACR-TIRADS and EU-TIRADS in pediatric age.

**Materials and methods:**

Subjects younger than 18 years of age with thyroid nodules greater than 0.5 cm observed in the 2000-2020 period were included.

**Results:**

Data from 200 subjects were collected. The overall ROM was 13%, rising to 26% if nodules with a diameter >1 cm were considered. Patients with a malignant nodule were more likely to have a higher EU-TIRADS score (p=0.03). Missed cancer diagnoses were 26.9%. Using the EU-TIRADS system, 40% of FNABs could have been avoided, while this scoring system would have resulted in FNAB being performed in 12% of cases where the assessment of ultrasound features would not recommend FNAB. Sensitivity, specificity, positive predictive value (PPV) and negative predictive value (NPV) were 73.1%, 57.1%, 73.1%, and 50%, respectively. Even considering the ACR-TIRADS, a higher score correlated with a higher ROM (p<0.001). This system missed 6 diagnoses of cancer (23.1%). Using the ACR-TIRADS system, 45.3% of FNABs could have been avoided, while FNAB should have been performed in 12% of cases where it was not recommended by ultrasound characteristics. Sensitivity, specificity, PPV and NPV were 76.9%, 50%, 76.9%, and 42.9%, respectively.

**Conclusion:**

The present study confirms the correspondence of the EU-TIRADS and ACR-TIRADS categories with respect to malignancy but indicates not entirely satisfactory performance compared to FNAB alone. However, the use of the two TIRADS systems should be encouraged in multicentre studies to increase their performance and establish paediatric-specific points in the scoring criteria.

## Introduction

Nodular thyroid disease in paediatric age has a lower prevalence (0.2-5.1%) than in adulthood (1-10%) (1–3), but the main difference between paediatric and adult age lies in the rate of malignancy (ROM) (16-26% vs 5-10%) (4–6). The most important risk factors for the development of thyroid cancer include underlying thyroid disease, radiation exposure, previous malignancy, family history, young age, male gender, and genetic predisposition (1–21).

Nodule size >1 cm, hypoechoic pattern, intranodal vascularization or microcalcifications, irregular edges and neck lymph nodes are the main ultrasound features that indicate malignancy (5, 22–29). Once suspicious ultrasound features are present, fine needle aspiration biopsy (FNAB) is required to determine the cytological category, identified based on the most widely used cytological classifications, namely the Bethesda System for Reporting Thyroid Cytopathology (BSRTC), the British Thyroid Association (BTA) and, in Italy, the Guidance of the Italian Society of Anatomic Pathology and Cytology (SIAPEC) (30–32). The cytological category assignment leads to different clinical management that includes clinical-radiological follow-up or surgery, with some differences between these classifications. Despite the differences, all agree on a higher ROM in paediatric age for all categories, especially for indeterminate nodules (6, 33, 34).

Based on the ultrasound features, the European Thyroid Association and the American Radiology College have established guidelines for Thyroid Imaging, Reporting and Data System (EU-TIRADS and ACR-TIRADS respectively) for risk stratification of malignancy of thyroid nodules. Once the TIRADS category is assigned, both guidelines determine whether to perform FNAB or adopt an active surveillance strategy, depending primarily on the size of the nodule (35, 36). The main reasons that led to the definition of these guidelines were the need to standardize the ultrasound description of thyroid nodules as much as possible, provide selection criteria to perform FNAB, avoid unnecessary procedures, and provide clinicians with an additional tool for the management of thyroid nodules, especially in the category of the indeterminate cytology.

Most existing studies evaluating ACR-TIRADS in adulthood have established that the score is useful for managing thyroid nodules and reducing the number of unnecessary FNAB procedures, while there are some concerns about its reliability for the evaluation of nodules with indeterminate cytology (37–51). Among the different TIRADS classification systems, ACR-TIRADS has been indicated as the most accurate classification system for identifying high-risk nodules and preventing most unnecessary FNABs (35, 36, 52–60), although in 10.2- 96 20% of cases a failure to diagnose malignancy has been reported (46, 55, 61).

To improve the diagnostic performance of ACR-TIRADS, some Authors have indicated additional nodules features or PET activity as risk factors (61, 62). Many efforts have been made to evaluate the diagnostic performance of ACR-TIRADS also in paediatric age (63). Its performance has been mostly defined as suboptimal, with a higher rate of cancers missed than in adulthood, up to 25% of cases (63, 64), suggesting that FNAB should be performed in all the 4 and 5 ACR-TIRADS categories (65–71). EU-TIRADS has also been considered a useful tool for physicians managing adults with thyroid nodules, although its performance should be improved and a FNAB should be performed in all nodules assessed as EU-TIRADS ≥4 (50, 72–75). Considering the EU-TIRADS, the rate of missed diagnosis of cancer is higher than that found in ACR-TIRADS, reaching up to 37.7% of cases (55, 72–79).

The purpose of this retrospective study is to evaluate the diagnostic performance of ACR-TIRADS and EU-TIRADS in risk stratification of paediatric thyroid nodules and determine whether extensive use of these tools can help the paediatric endocrinologist better manage thyroid nodules in pediatric age.

## Materials and methods

The study included all subjects under the age of 18 with thyroid nodules greater than 0.5 cm followed at the Tertiary Center of Paediatric Endocrinology of the Regina Margherita Children’s Hospital in Turin in the period 2000-2020. Patients with nodules less than 0.5 cm in diameter and with suspicious characteristics were also initially considered. However, none of these were then included in the study as no malignant features were found in any of these nodules. After approval by the Institute’s Ethical Committee, clinical, laboratory and radiographic data were collected from electronic medical records. All patients underwent thyroid ultrasound evaluation, which assessed the diameter of the nodule and the ultrasound pattern; they were therefore classified as anechoic, hypoechoic, isoechoic, hyperechoic, or mixed nodules. All lymph node changes were then recorded, such as rounded swollen shape, irregular margins, increased size, absence of echogenic hilum, heterogeneous echo pattern, presence of calcifications or cystic areas, and irregular vascularization. Patients undergoing multiple ultrasound monitoring were considered as a single case. In patients with multiple nodules, the largest nodule was considered.

All ultrasound evaluations were performed in the same institution and the images were retrospectively evaluated by two independent radiologists blinded for the outcome. The TIRADS category was indicated according to both EU-TIRADS and ACR-TIRADS. Patients with inadequate ultrasound images to correctly assess the TIRADS category were excluded. In case of nodules >1 cm or suspicious features of malignancy on ultrasound evaluation, a cytological sample was obtained by fine needle aspiration biopsy (FNAB) within one month of the ultrasound finding. Histological specimens were also obtained from subjects undergoing lobectomy or total thyroidectomy. All specimens were evaluated by a single pathologist.

Statistical analysis and graphs construction were performed using Graphpad 7 (GraphPad Software, La Jolla, CA, USA). Sensitivity (number of true positives divided by the sum of true positives and false negatives), specificity (number of true negatives divided by the sum of true negatives and false positives), positive predictive value (number of true positive divided by the sum of true positive and false positive), negative predictive value (number of true negative divided by the sum of true negative and false negative) and diagnostic accuracy (sum of true positives and true negatives divided by the samples’ number) were calculated based on the results of patients undergoing both FNAB and surgery. Differences between groups were established by t test to compare mean values of continuous variables. The calculations were considered statistically significant when the P-value was <0.05. Cohen’s kappa coefficient was calculated to measure the inter-rater reliability among the radiologists assigning the TIRADS score.

## Results

We collected clinical, laboratory and ultrasound retrospective data from 200 subjects (119 females and 81 males) aged less than 18 years with thyroid nodules (**Table 1**). The observed overall rate of malignancy (ROM) was 13% (26/200 malignant nodules), which rose to 26% if nodules with a diameter >1 cm were considered. The mean age at diagnosis was 11.6 years (range 2-18), with a mean follow-up of 8.6 years. The ratio of female to male was 1.47 and dropped to 1.27 considering the malignant nodules.

**Table 1:** Clinical, biochemical and US features of all subjects (N=200) with thyroid nodules.

Clinical, biochemical and US features		All (n = 200)	Benign (n = 174)	Malignant (n = 26)	p
Age at diagnosis (years)		12 (2–18)	12 (2-18)	12.9 (7-17.1)	0.22
Gender (M/F)	M	81	70	11	0.65
	F	119	104	15	
Thyroid disease familiarity		108	96	12	0.66
Radiation exposure		28	22	6	0.1
TSH (mcUI/ml)		2.01 (0.1-5.3)	1.94 (0.1-4.9)	2.56 (0.8-5.3)	**0.01**
fT4 (pg/ml)		11.5 (1.05-134)	11.5 (1.05-13.4)	12 (7.3-15.3)	0.55
fT3 (pg/ml)		4.05 (2.3-5.8)	4 (3.06-5.8)	4.09 (2.3-4.6)	0.18
Thyroid antibodies positivity		69	53	16	0.8
Nodule localization	Left lobe	91	86	5	**0.01**
	Right lobe	92	75	17	
	Bilateral	17	13	4	
Major nodule diameter (mm)		9 (8-60)	8 (8-10)	24 (7-60)	**<0.0001**
Echoic pattern	Hypoechoic	121	101	20	0.24
	Hyperechoic	14	14	0	
	Isoechoic	20	18	2	
	Anechoic	17	17	0	
	Mixed	28	24	4	
Intranodal vascularity		61	46	15	**0.003**
Intranodal calcifications		23	15	8	**0.009**
Lymph node involvement		11	1	10	**<0.0001**

Bold is for statistical significant.

Regarding risk factors such as age, gender, family history of thyroid diseases, positive thyroid antibodies and radiation exposure for cancer previously treated with radiotherapy, no difference was observed between benign and malignant nodules.

All subjects had normal levels of fT4 and fT3, but the TSH level was significantly lower in subjects with a benign nodule than in subjects with a malignant nodule *(p=0.01).*

Bilateral and right lobe involvement was associated with a higher malignancy rate than left lobe localization (23.6% vs 18.5% vs 5.5% malignancy rate, respectively, *p=0.01*), as also observed for intranodal vascularization and calcification (*p=0.003* and *p=0.009*, respectively), and lymph node involvement (*p<0.0001*). A larger nodule diameter was significantly more present in the malignant nodule than in the benign nodule group (mean diameter 24 mm vs 8 mm, respectively, *p=<0.001*). The echogenic pattern was not related to ROM.

FNAB was performed based on nodule size and ultrasound features in 75/200 (37.5%) of subjects, including 7 TIR1 (9.3%), 4 TIR1c (5.3%), 22 TIR2 (29.3%), 14 TIR3a (18.7%), 9 TIR3b (12%), 3 TIR4 (4%) and 16 TIR5 (21.4%).

Surgery was performed in 40/200 (20%), with a total malignancy rate of 65% (0% for the TIR1-TIR3a, 77.8% for the TIR3b and 100 % for the TIR4-TIR5 categories) as shown in **Table 2**.

**Table 2:** Cytological, histological data, malignancy rate and FNAB accuracy for each cytological category.

SIAPEC category	Nr	Surgery	Outcome	ROM	FNAB Accuracy
TIR1	7 (9.3%)	–	All benign	0%	–
TIR1C	4 (5.3%)	1	All benign 1 ST for cystic nodule and dysphagia	0%	100%
TIR2	22 (29.3%)	5	All benign 5 TTs for important MNS	0%	100%
TIR3a	14 (18.7%)	1 TT 5 ST	6 patients with benign histology 8 patients still undergoing ultrasound/FNAB follow-up	0%	100%
TIR3b	9 (12%)	7 TTs 2 STs	All subjects underwent surgery 7 patients with malign histology 2 STs with NS histological diagnosis	77.8%	77.8%
TIR4	3 (4%)	3 TTs	All malign PTC histological diagnosis in all subjects	100%	100%
TIR5	16 (21.4%)	16 TTs	All malign PTC histological diagnosis in all subjects	100%	100%
Total	75	40		65%	95%

ROM, rate of malignancy; ST, subtotal thyroidectomy; TT, total thyroidectomy; MNS, multinodular struma; NS, nodular struma; PMC, papillary micro carcinoma; PTC, papillary thyroid carcinoma; FTC, follicular thyroid carcinoma.

Cohen’s kappa coefficient among the radiologists assigning the TIRADS score was 0.85. The correlation between cytological categories after FNAB and the EU-TIRADS score is represented in **Table 3**. Patients with a malignant nodule were more likely to have a higher EU-TIRADS score ***(p=0.03)***. If all nodules are considered, the most frequently assigned category was EU-TIRADS 4 (53%, ROM 16.9%), followed by EU-TIRADS 2 (18.5%, ROM 0%), EU-TIRADS 3 (18%, ROM 8.3%) and EU-TIRADS 5 (10.5%, ROM 23.8%). Nodules with cytological determination were mainly assigned to EU-TIRADS 4 (46.7%), with ROM up to 48.6%, followed by EU-TIRADS 3 (30.1%, ROM 21.7%), EU-TIRADS 2 (14.7%, ROM 0%) and EU-TIRADS 5 (8%, ROM 83.3%).

**Table 3:** Correlation between cytological categories and EU-TIRADS score.

All nodules									
	All (n = 200)		Benign (n = 174)		Malign (n = 26)			ROM	p
EU-TIRADS 2	37		37		–			0%	p=0.03
EU-TIRADS 3	36		33		3			8.3%	
EU-TIRADS 4	106		88		18			16.9%	
EU-TIRADS 5	21		16		5			23.8%	
**Nodules with cytologic determination**									
		SIAPEC cytologic category after FNAB						ROM	Score accuracy on ROM
		TIR 1/1C (n=11)	TIR 2 (n=22)	TIR 3a (n=14)	TIR 3b (n=9)	TIR 4 (n=3)	TIR 5 (n=16)	
EU-TIRADS 2 (n=11)	FNAB	–	–	–	–	–	–	0%	100%
	NO FNAB	4	3	4	–	–	–
EU-TIRADS 3 (n=23)	FNAB	4	1	6	–	–	–	21.7%	78.2%
	NO FNAB	–	3	4	–	–	5
EU-TIRADS 4 (n=35)	FNAB	–	8	–	8	3	7	48.6%	100%
	NO FNAB	3	6	–	–	–	–
EU-TIRADS 5 (n=6)	FNAB	–	1	–	1	–	2	83.3%	66.7%
	NO FNAB	–	–	–	–	–	2

The correlation between the FNAB categories and the ACR-TIRADS system score is represented in **Table 4**. Higher scores correlated with higher ROMs *(p<0.001)*. Most nodules were classified as ACR-TIRADS 4 (54.5%, ROM 15.6%), followed by ACR-TIRADS 3 (24.5%, ROM 4.1 %), ACR-TIRADS 1 (10%, ROM 0%), ACR-TIRADS 5 (6.5%, ROM 53.8%) and ACR-TIRADS 2 (4.5%, ROM 0%). When only nodules with cytological determination were considered, the category assigned in most cases was ACR-TIRADS 4 (42.7%, ROM 75%), followed by ACR-TIRADS 5 (17.3%, ROM 53.8 %), ACR-TIRADS 3 (16%, ROM 16.7%), ACR-TIRADS 1 (13.3%, ROM 0%) and ACR-TIRADS 2 (10.7%, ROM 0%).

**Table 4:** Correlation between cytological categories and ACR-TIRADS score.

All nodules											
	All (n = 200)			Benign (n = 174)			Malign (n = 26)			ROM	p
ACR TIRADS 1	20			20			–			0%	p<0.0001
ACR TIRADS 2	9			9			–			0%	
ACR TIRADS 3	49			47			2			4.1%	
ACR TIRADS 4	109			92			17			15.6%	
ACR TIRADS 5	13			6			7			53.8%	
**Nodules with cytologic determination**											
		SIAPEC cytologic category after FNAB									Score accuracyon ROM
		TIR 1/1C (n=11)	TIR 2 (n=22)		TIR 3a (n=14)	TIR 3b (n=9)		TIR 4 (n=3)	TIR 5 (n=16)	ROM
ACR-TIRADS 1 (n=10)	FNAB	–	–		–	–		–	–	0%	100%
	NO FNAB	5	4		1	–		–	–	
ACR-TIRADS 2 (n=8)	FNAB	–	–		1	–		–	–	0%	100%
	NO FNAB	2	4		1	–		–	–
ACR-TIRADS 3 (n=12)	FNAB	–	–		1	–		–	–	16.7%	83.3%
	NO FNAB	3	5		1	–		–	2
ACR-TIRADS 4 (n=32)	FNAB	–	4		4	9		3	3	75%	87.5%
	NO FNAB	1	3		1	–		–	4
ACR-TIRADS 5 (n=13)	FNAB	–	2		2	–		–	7	53.8%	100%
	NO FNAB	–	–		2	–		–	–

Based on the EU-TIRADS score, missed cancer diagnoses would have occurred in 7 cases (26.9%), with 5 nodules classified in category 3 and 2 nodules in category 5 (**Table 5**). All nodules in category 3 were < 20 mm and in category 5 < 10 mm. All missed diagnoses were assigned to the TIR5 cytological category. Using the EU-TIRADS system, 40% (30/75) of the FNABs performed could have been avoided, while this scoring system would have led to perform a FNAB in 12% (15/125) of the cases in which the assessment of the ultrasound features would not have recommended FNAB. Sensitivity, specificity, positive predictive value, and negative predictive value based on histological outcome were 73.1%, 57.1%, 73.1%, and 50% respectively.

**Table 5:** Risk stratification performance of EU-TIRADS and ACR-TIRADS in paediatric age.

	Sensibility	Specificity	PPV	NPV	Accuracy on ROM	Missed cancer	Avoided FNAB	AddictiveFNAB
EU-TIRADS	73.1%	57.1%	73.1%	50%	90.7%	7/26	30/75	15 (12%)
ACR-TIRADS	76.9%	50%	76.9%	42.9%	92%	6/26	34/75	15 (12%)
SIAPEC FNAB	100%	85.7%	92.9%	100%	95%	–	–	–

With the ACR-TIRADS system, cancer diagnosis would have been lost in 6 cases (23.1%), with 2 nodules assigned to ACR-TIRADS category 3 and 4 nodules to ACR-TIRADS category 4. All missed diagnoses were classified cytologically as TIR5. Using the ACR-TIRADS system, 45.3% (34/75) of the FNABs performed could have been avoided; on the other hand, a FNAB was indicated in 12% (15/125) of cases in which it was not recommended by the evaluation of the ultrasound features. Sensitivity, specificity, positive predictive value, and negative predictive value based on histological outcome were 76.9%, 50%, 76.9%, and 42.9%, respectively. The sensitivity and specificity of FNAB based on histological outcome for all categories were 100% and 85.7%, respectively, while PPV and NPV were 92.9% and 100%, respectively. Considering the high ROM of the nodules within the TIR3b category, all nodules classified in TIR3b were considered cytologically malignant. Compared to FNAB, ROM accuracy was lower for both EU-TIRADS and ACR-TIRADS (95% vs 90.7% and 92% respectively).

## Discussion

Thyroid nodules in paediatric age have a lower prevalence than in adulthood, but greater ROM (1–7). Considering only nodules >1 cm, the ROM rate of our cohort was 26%, in line with previous published studies. The overall ROM rate was 13%, probably underestimated as most patients did not have suspicious ultrasound features leading to FNAB.

The behaviour of pediatric thyroid cancer is different from that of adults, with higher rates of extrathyroid extension and disease recurrence, but much better prognosis and survival rates; to date their management therefore remains challenging. Giving the invasiveness of FNAB, to avoid unnecessary procedures and anxiety for children and their parents, the best follow-up strategy should include this procedure only when strictly necessary, in presence of certain clinical and ultrasound features. The most important, reported by the current guidelines for adults, is the size of the nodule greater than 1 cm. Other features include intranodal calcification or vascularization, lymph node involvement, marked hypoechoic pattern, bilateral or right lobe localization of the nodule, poorly defined nodule margins and some clinical risk factors, particularly radiation exposure for cancer treatment, increased TSH values, young age and male gender. In our cohort, TSH levels were correlated to malignancy, as previously reported (6, 26). Considering the child’s body size and the presence of microcarcinomas, in presence of multiple risk factors FNAB should be performed even if the nodule size is smaller than 1 cm (1–7).

For both the paediatric and adult populations, numerous efforts have been made to improve the selection criteria that lead clinicians to perform FNAB. To standardize the ultrasound description of thyroid nodules as much as possible and better select candidates for FNAB, the European Thyroid Association and the American Radiology College have established guidelines for Thyroid Imaging, Reports and Data System. Despite several limitations of both scoring system, previous studies in paediatric and adult cohorts have encouraged their use to increase the available data that can improve their performance. EU-TIRADS categories have been observed to be related to thyroid nodules malignancy, although the performance of such system should be improved and therefore a FNAB is currently recommended in all EU-TIRADS ≥4 nodules (50, 72–75), as cancer underdiagnosis rate rises to 37.7% (55, 72–80). The sensitivity, specificity, positive predictive value (PPV) and negative predictive value (NPV) of EU-TIRADS in adulthood are between 70.6-83.5%, 51.2-94.1%, 11.8-76.1% and 85.4-94.9%, respectively (62, 73–75, 77, 78). The performance of EU-TIRADS in paediatric age has been evaluated in a few studies that showed lower efficacy than in adults, with sensitivity, specificity, PPV and NPV ranging between 41.7-100%, 25-75.9%, 41.7-44%, 75.9-100% respectively (63, 78, 79). The data from our study confirm the significant correlation of the EU-TIRADS category with malignancy. Sensitivity, specificity, PPV and NPV were 73.1%, 57.1%, 73.1% and 50 %, respectively, showing an underestimation of malignant lesions and a low ability to detect histologically determined benign nodules, which do not require FNAB. Lost cancer diagnoses in our cohort were 26.9%, while 40% of FNABs could have been avoided and 12% of patients who were not selected for needle-biopsy should have undergone FNAB.

Most existing studies evaluating ACR-TIRADS in adulthood have determined that the system score is useful for managing thyroid nodules and reducing the number of unnecessary FNAB, while some concerns remain about its reliability in evaluating nodules of indeterminate cytology (37–51). Among the different TIRADS, ACR-TIRADS was ranked as the best performing classification for identifying high-risk nodules and unnecessary FNABs (35, 36, 52–60), although a missed malignancy diagnosis occurred in 10.2-20% of cases in which ACR-TIRADS have not indicated the need for FNAB (46, 55, 61). The combined sensitivity and specificity of ACR-TIRADS in adults were 89% and 70%, respectively (61). Many efforts have been made to evaluate the diagnostic performance of ACR-TIRADS also in paediatric age (63), mostly defined as suboptimal, with a rate of up to 25% of undiagnosed cancers, higher than that of adulthood (63, 64), which suggests performing FNAB in all ACR-TIRADS categories 4 and 5 (65–71). Sensitivity, specificity, PPV and NPV in the paediatric age group vary between 70-75%, 64-92.3%, 21.8-83.3% and 64-97.2%, respectively (65, 67–71). In our study, the performance of ACR-TIRADS was similar to that indicated by the literature for sensitivity, but the specificity was lower, and the cancer underdiagnosis rate higher (23.1%). Using ACR-TIRADS, 45.3% of FNAB could have been avoided, while 12% of unselected patients would have had to undergo FNAB.

Considering the two scoring systems, ACR-TIRADS performed better than EU-TIRADS as also observed in previous studies (52–60). The number of potentially avoidable FNABs was substantial, although malignant nodules were underestimated and the performance of both scores to avoid FNAB in definitive benign nodules was not satisfactory. The interpretation of FNAB results according to the SIAPEC classification has a significantly greater risk stratification capacity, with a sensitivity of 100%. This is mainly due to the interpretation of the indeterminate category TIR3b as cytologically malignant, with consistently high ROM (77.8%), while the ROM observed in the indeterminate category TIR3a was 0%. This result differs from the BSRTC system which assigns similar ROMs in the indeterminate grouped categories Bethesda III and IV.

Despite the limitations of TIRADS scores, we confirm that their use should be encouraged to improve their performance and have an additional tool in the management of paediatric thyroid nodules. The main limitation of TIRADS in children is the criterion of the size as a determinant for the execution of FNAB. We must be aware that the current guidelines have been established for adults and are not at all suitable for children, especially considering their body size. The EU-TIRADS score does not include lymph node involvement in the score but indicates the need for FNAB in case of suspicious ultrasound features; intranodal vascularization, described as a risk factor for malignancy, is also not included in the EU or in the ACR-TIRADS. Bilateral and right lobe localization should also be considered in the final score. To improve the diagnostic performance of ACR-TIRADS, some authors have indicated additional characteristics as risk profiles or PET activity (61, 62). The association of ultrasound data with clinical data could be an additional aid to performance improvement. The final score could also include an age <10 years, male gender, previous radiation exposure for cancer treatment, a higher TSH level, as well as a familial history or genetic predisposition to thyroid cancer (1–5).

The present study has several limitations. The retrospective nature of the study limits the statistical power of the data analysis. The number of histologically and cytologically determined malignant nodules is limited due to the low prevalence of pediatric thyroid nodules and restrictive criteria for FNAB, which can lead to underestimation, despite the case series being recruited in a tertiary centre of Paediatric Endocrinology over a 20-years period.

In conclusion, in the present study the correlation of the EU-TIRADS and ACR-TIRADS categories with malignancy was confirmed, even if their performance was not entirely satisfactory compared to FNAB alone. However, their use should be encouraged within multicentre studies, to increase the performance of both TIRADS systems and to allow for an update of the scoring criteria, including pediatric-specific points.

## Data availability statement

The raw data supporting the conclusions of this article will be made available by the authors, without undue reservation.

## Ethics statement

The studies involving human participants were reviewed and approved by Ethics Committee of the City of Health and Science University Hospital of Turin. Written informed consent to participate in this study was provided by the participants’ legal guardian/next of kin.

## Author contributions

GT and JM contributed to the study concept, the statistical analysis and to the first draft of manuscript. MS contributed to the data collection and literature research. FQ and LS contributed to the study concept and the revision con the final version of the manuscript. All authors contributed to the article and approved the submitted version.

## Conflict of interest

The authors declare that the research was conducted in the absence of any commercial or financial relationships that could be construed as a potential conflict of interest.

## Publisher’s note

All claims expressed in this article are solely those of the authors and do not necessarily represent those of their affiliated organizations, or those of the publisher, the editors and the reviewers. Any product that may be evaluated in this article, or claim that may be made by its manufacturer, is not guaranteed or endorsed by the publisher.
